# Antibiotics and Metabolic Dysregulation After Surgery: Exploring an Overlooked Connection

**DOI:** 10.2174/0115733998425015260116113449

**Published:** 2026-03-02

**Authors:** Dinesh Kumar, Vrinda Gupta, Nicky Kumar Jaiswal, Rajni Tanwar

**Affiliations:** 1 School of Pharmacy, Desh Bhagat University, Mandi Gobindgarh, Punjab, India;; 2 Department of Pharmaceutical Sciences, Guru Jambheshwar University of Science and Technology, Hisar, Haryana, India

**Keywords:** Antibiotics, gut microbiota, hyperglycemia, insulin resistance, hypertension, dysbiosis

## Abstract

Hypertension and hyperglycemia are common in patients without metabolic disease even after surgery and are generally considered to have been caused by surgical stress and inflammation. Nonetheless, in the case of long-term or repeated exposure to perioperative antibiotics, this can be a hidden cause of disturbances. Broad-spectrum antibiotics may interfere with gut microbiota, decrease short-chain fatty acid synthesis, and impair mitochondrial function and vascularity, thereby facilitating the development of insulin resistance and metabolic instability. Observational cohort evidence associates cumulative exposure to antibiotics with the risk of developing type 2 diabetes, whereas experimental models show that antibiotics cause glucose intolerance *via* the mechanism of dysbiosis. There are also clinical observations that, in certain patients, suitable parameters of the metabolic condition return to normal only on removal of antibiotics. These mechanisms can interact with surgical stress, especially in metabolically vulnerable individuals. This hypothesis-generating editorial demonstrates the need to explore antibiotic-related metabolic changes during postoperative care and to implement microbiome-informed interventions in perioperative practice and antibiotic stewardship.

## 1 INTRODUCTION

Given the recent recognition of antibiotic-associated dysbiosis as a contributor to the pathogenesis of insulin resistance and hyperglycemia, this editorial examines postoperative metabolic abnormalities associated with antibiotic use and their potential role in the development of new-onset diabetes. Clinical observations indicate that patients postoperatively report an increase in blood pressure and blood glucose, though they have no previous history of metabolic disease [1]. Although surgical stress, inflammation, and restricted mobility are proven factors of transient metabolic imbalance, some cases exhibit the restoration of metabolic parameters upon antibiotic discontinuation [2]. Surgery, such as gastrointestinal surgery, may modify body metabolism by altering nutrient absorption and gut signaling pathways [3]. To illustrate, obesity bypass and sleeve surgeries can rapidly alter glucose and incretin levels. On the contrary, colorectal surgery could lead to gut dysbiosis, which can down-regulate metabolism [4]. The present hypothesis, however, aims to describe why prophylactic antibiotics have been used universally in surgical specialties like orthopedics and cardiothoracic surgery and hypothesizes that antibiotics can influence dysbiosis, thus magnifying the metabolic impact of surgery [5].

Notably, despite antidiabetic and antihypertensive medications being effective in maintaining blood sugar and blood pressure levels in patients at the initial stage of treatment, both tend to increase later, as the evidence of specific antibiotics in the body persists. It may be that the use of antibiotics in the post-surgery period is linked to slower metabolic issues. Antibiotics, especially those with broad-spectrum activity, administered post-surgery can modify metabolic parameters. Many plausible biological explanations support this hypothesis [3]. Even with the best surgical practices, some patients who do not have a pre-existing metabolic illness often develop postoperative hyperglycemia and hypertension, raising the question of other unknown factors beyond surgical stress and inflammation [4]. It is well known that broad-spectrum antibiotics disrupt the gut microbiome, reducing the production of short-chain fatty acids (SCFAs), which are very important for regulating glucose and endothelial well-being [5]. SCFA depletion also contributes to insulin resistance and vascular stiffness, predisposing the patient to metabolic instability post-surgery [6]. Moreover, these disturbances are enhanced by antibiotic-induced mitochondrial stress and increased inflammatory signaling [7]. Evidence from both human and animal studies supports this connection. Accumulating evidence from large cohort studies indicates that long-term or repeated antibiotic exposure is associated with a modest but consistent increase in the incidence of type 2 diabetes. Individuals receiving multiple antibiotic courses exhibited higher adjusted hazard ratios compared with those with minimal or no exposure, suggesting a dose-response relationship, where in some individuals, none were prescribed for the short term [8]. Hyperglycemia can occur in some patients who receive certain antibiotics and may have previously had stable glycemic control, making antibiotics that can cause metabolic disturbances in susceptible individuals stand out. Antibiotic dysbiosis may be an essential factor in understanding host metabolic changes and is most relevant in the context of added surgical stress and perioperative inflammation [9].

For example, a population-based cohort study reported a higher incidence of type 2 diabetes in patients with repeated antibiotic exposure. At the same time, murine models demonstrated antibiotic-induced glucose intolerance mediated *via* gut microbiota disruption. Another matter to consider is when antibiotics alter the metabolism or absorption of antihypertensive and antidiabetic drugs, potentially reducing their effects and requiring higher or more frequent doses [10]. There may be more of these cases now than we are aware of. Since antibiotics are regularly prescribed after surgery in many different fields and millions of operations are carried out around the world each year, the impact of a small but regular metabolic side effect could be significant for many people [11]. Across a variety of clinical settings, postoperative antibiotics are prescribed and then either increased in dosage or continued for longer than suggested by guidelines [12]. This practice might be viewed as overuse or abuse of antibiotics. Such practices of excessive, indiscriminate antibiotic use are more likely to provoke gut microbiota imbalance, mitochondrial dysfunction, and chronic inflammation, thus establishing a pathway to explain the routine use of postoperative antibiotics and the associated metabolic disorders that are frequently observed after surgery [13].

In elderly patients and those who already have metabolic syndrome, small changes in blood pressure or blood sugar can be dangerous, causing wound healing delays, an increased risk of infection, heart problems, or a requirement to stay hospitalized for an extended period. Furthermore, significant metabolic changes may result from antibiotic-induced disruptions in gut balance, but these are currently under-researched [14]. Given the widespread perioperative use of antibiotics, even modest metabolic consequences may impact recovery and long-term outcomes, particularly among elderly and metabolically vulnerable patients. Targeted research, including microbiome and metabolomic analyses, is essential to clarify these associations and identify preventive strategies, such as antibiotic stewardship and the adjunctive use of prebiotics or probiotics [15].

## CONCLUSION

Based on the findings, it can be concluded that further research into antibiotic-related metabolic effects may improve postoperative care and patient recovery trajectories [16].
